# Early evolocumab improves lipid profile, inflammation and short-term outcomes in non-revascularized CHIP-NSTEACS patients: a retrospective cohort study

**DOI:** 10.3389/fcvm.2026.1901995

**Published:** 2026-08-12

**Authors:** Wenlong Jiang, Mei Liu, Xiaoshuan Xiong, Jing Cheng, Fangzhou Cheng

**Affiliations:** Department of Cardiovascular, Shenzhen Yantian District People’s Hospital, Shenzhen, China

**Keywords:** CHIP, evolocumab, inflammation, non-revascularization, NSTEACS, PCSK9 inhibitor

## Abstract

**Objective:**

To evaluate the lipid-lowering efficacy, anti-inflammatory activity, and short-term prognostic benefit of early evolocumab plus statin in complex high-risk indicated non-revascularized non-ST-elevation acute coronary syndrome (CHIP-NSTEACS) patients.

**Methods:**

This single-center retrospective cohort study consecutively enrolled 186 CHIP-NSTEACS patients hospitalized from January 2022 to December 2024. Patients were divided into an observation group (high-intensity statin + evolocumab initiated within 48 h of admission, *n* = 92) and a control group (statin monotherapy, *n* = 94). Inverse probability of treatment weighting (IPTW) was used to balance baseline covariates. Longitudinal changes in lipid profiles and high-sensitivity C-reactive protein (hs-CRP) were analyzed using repeated-measures ANOVA. The primary endpoint was 3-month major adverse cardiovascular events (MACE). Multivariate Cox regression and Fine-Gray competing risk models were used for survival analysis.

**Results:**

Baseline characteristics were well-balanced after IPTW (all standardized mean differences <0.1). At 3 months, LDL-C reduction was 61.3% in the observation group vs. 32.2% in the control group (*P* < 0.001), with target achievement rates (<1.8 mmol/L) of 89.1% vs. 27.7% (*P* < 0.001). Evolocumab also significantly reduced lipoprotein(a) and hs-CRP (48.7% vs. 26.1% at 1 month, *P* < 0.001). The 3-month MACE incidence was lower in the observation group (11.96% vs. 28.72%, *P* = 0.004), driven by reductions in non-fatal myocardial infarction, recurrent angina, and unplanned cardiac rehospitalization. Multivariate Cox regression identified early evolocumab as an independent protective factor against 3-month MACE (HR = 0.42, 95% CI: 0.25–0.71, *P* = 0.001). No significant between-group difference in adverse events was observed (5.43% vs. 4.26%, *P* = 0.715).

**Conclusion:**

Early evolocumab add-on therapy to high-intensity statin achieves potent lipid-lowering and anti-inflammatory effects, improves 3-month clinical outcomes, and has an acceptable safety profile in non-revascularized CHIP-NSTEACS patients. This strategy represents a promising optimized pharmacotherapy for this ultra-high-risk population.

**Limitations:**

Single-center retrospective design and short follow-up period limit generalizability.

## 1 Introduction

Non-ST-elevation acute coronary syndrome (NSTEACS) remains a leading contributor to cardiovascular-related hospitalization and premature mortality globally, driven by atherosclerotic plaque rupture, persistent vascular inflammatory activation, and substantial residual ischemic risk. A substantial subset of NSTEACS patients present with complex high-risk indicated patient (CHIP) phenotype, defined by multi-vessel coronary artery disease, left main involvement, severe calcified/tortuous coronary lesions, advanced age, and concurrent irreversible severe extra-cardiac organ dysfunction (1, 2). Although international guidelines recommend revascularization via percutaneous coronary intervention (PCI) or coronary artery bypass grafting (CABG), invasive revascularization is frequently abandoned owing to prohibitive procedural risks, critical comorbidities, or patient's informed refusal (3).

Non-revascularized CHIP-NSTEACS constitutes an understudied ultra-high-risk subgroup with exaggerated atherosclerotic plaque burden, sustained systemic inflammation, and suboptimal residual lipid control compared with revascularized NSTEACS counterparts (4). Conventional high-intensity statin monotherapy commonly fails to attain guideline-directed LDL-C targets and cannot sufficiently suppress persistent low-grade inflammation, resulting in excessive short-term MACE and recurrent hospital readmission (5).

Proprotein convertase subtilisin/kexin type 9 (PCSK9) inhibitor evolocumab is a cornerstone agent for intensive lipid-lowering in atherosclerotic cardiovascular disease (ASCVD) (6). Large randomized controlled trials including FOURIER and ODYSSEY OUTCOMES have validated its ability to rapidly lower LDL-C, stabilize vulnerable coronary plaque, mitigate inflammatory burden, and reduce long-term cardiovascular events predominantly among ACS patients receiving subsequent PCI (7, 8). However, real-world evidence focusing specifically on non-revascularized CHIP-NSTEACS cohorts remains scarce, and the short-term lipid-modulating, anti-inflammatory, and prognostic benefits of early in-hospital evolocumab initiation have not been fully validated in this unique high-risk population.

Study Hypothesis: Early evolocumab combined with high-intensity statin exerts superior lipid-lowering and anti-inflammatory effects and reduces 3-month MACE risk in non-revascularized CHIP-NSTEACS patients compared with statin monotherapy.

The present retrospective cohort study was designed to verify the above hypothesis, complement real-world clinical evidence for individualized intensive pharmacotherapy, and provide clinical reference for guideline optimization in non-revascularized CHIP-NSTEACS population.

## 2 Materials and methods

### 2.1 Ethical approval

This retrospective study was approved by the Ethics Committee of Shenzhen Yantian People's Hospital (Approval No. [2022-023]). Individual written informed consent was waived because all clinical data were fully anonymized before analysis, and all research procedures complied with the Declaration of Helsinki.

### 2.2 Study population

Consecutive hospitalized patients diagnosed with CHIP-NSTEACS between January 2022 and December 2024 were retrospectively screened. CHIP-NSTEACS diagnostic criteria: (1) NSTEACS confirmed by typical angina symptoms, dynamic ST-T changes on 12-lead ECG, and elevated serum cardiac troponin; (2) coronary angiography verified ≥2-vessel disease or left main coronary stenosis with SYNTAX score ≥22; (3) no PCI/CABG performed during index hospitalization due to advanced age, severe organ insufficiency, excessive surgical risk, or voluntary refusal.

Inclusion criteria:

1. Consistent with international diagnostic criteria of NSTEACS.
2. CHIP angiographic/clinical features without in-hospital revascularization.
3. Receiving standardized high-intensity statin: atorvastatin 40 mg once nightly or rosuvastatin 20 mg once nightly.
4. Complete inpatient laboratory, clinical, and follow-up endpoint datasets available.

Exclusion criteria:

1. Underwent PCI/CABG within 72 h of symptom onset.
2. Severe hepatic/renal impairment (ALT/AST > 3× upper limit of normal, eGFR < 30 mL/min/1.73 m^2^).
3. Concurrent active acute infection, malignant tumor, autoimmune, or severe hematological disorders.
4. Confirmed allergic history to statins or evolocumab.
5. Receiving ezetimibe or other non-statin lipid-lowering therapies (e.g., fibrates, omega-3 fatty acids) prior to admission or during the follow-up period.
6. Lost to follow-up or incomplete outcome documentation.

**Figure 1:**
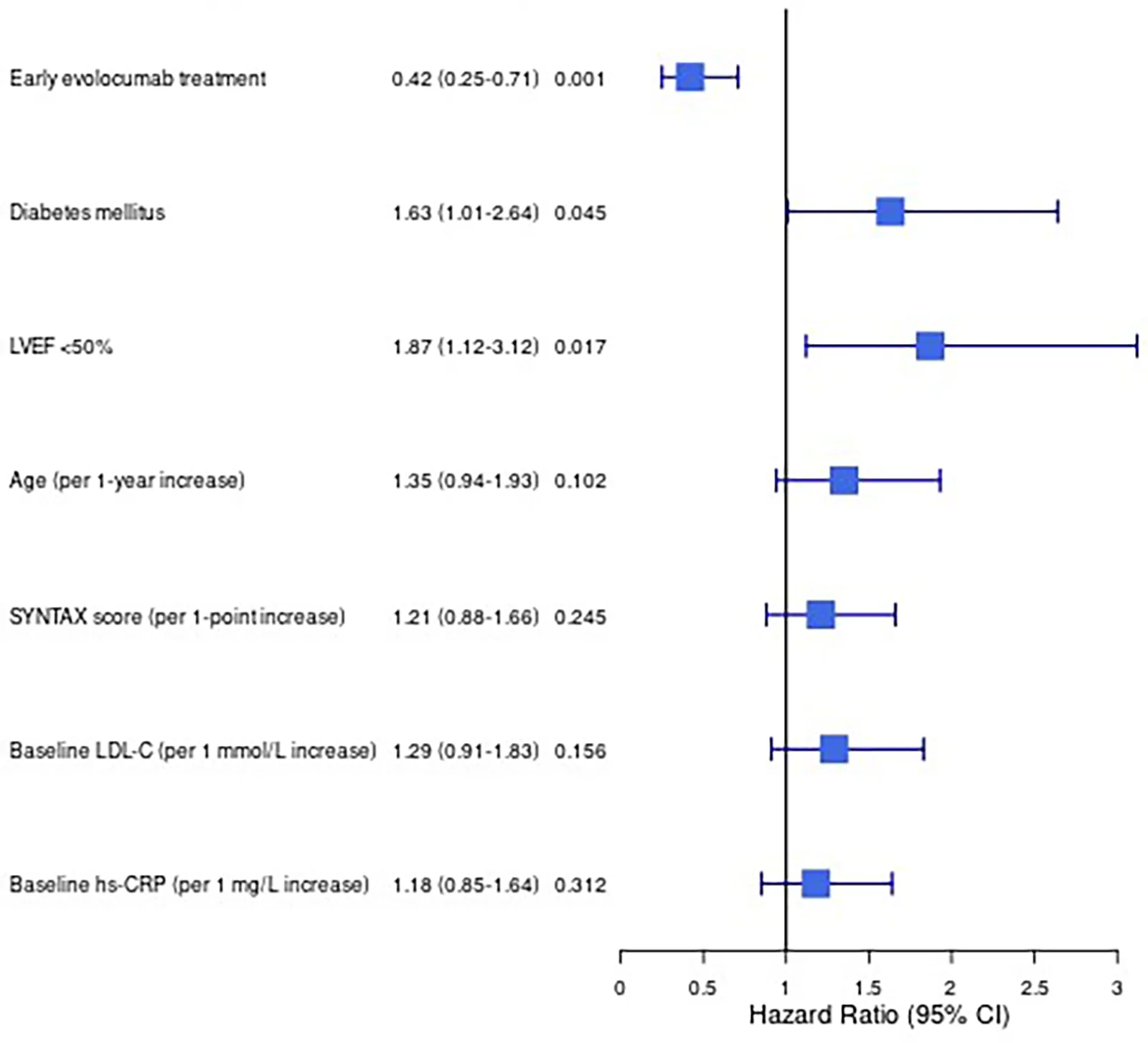
Forest plot of multivariate Cox proportional hazards regression analysis identifying independent predictors of 3-month major adverse cardiovascular events (MACE) in non-revascularized CHIP-NSTEACS patients after inverse probability of treatment weighting adjustment. Hazard ratios (HR) and corresponding 95% confidence intervals (CIs) are displayed for all candidate covariates. The vertical dashed reference line at HR = 1.0 denotes no prognostic effect; horizontal bars represent 95% CIs for each variable. HR values < 1 indicate protective factors, while HR values > 1 indicate adverse risk factors. LVEF, left ventricular ejection fraction; LDL-C, low-density lipoprotein cholesterol; hs-CRP, high-sensitivity C-reactive protein.

After initial screening, 186 eligible patients were ultimately enrolled (Figure 1).

### 2.3 Grouping and standardized therapeutic regimens

Patients were grouped based on whether subcutaneous evolocumab was initiated within 48 h after admission. The decision to prescribe evolocumab was made by the treating cardiologist as part of a multidisciplinary heart team assessment and was not based on a predefined LDL-C threshold. Rather, it was based on the clinical judgment of the ultra-high-risk nature of the patient, specifically the diagnosis of CHIP-NSTEACS with ineligibility for revascularization, in conjunction with other high-risk features such as elevated inflammatory biomarkers, diabetes, or prior cardiovascular events. This real-world treatment allocation reflects routine clinical practice. To rigorously control for potential confounding by indication, we employed Inverse Probability of Treatment Weighting (IPTW), which accounts for measured baseline covariates that may have influenced treatment decisions:

Observation group (*n* = 92): High-intensity statin + evolocumab 140 mg subcutaneous injection every 2 weeks.

Control group (*n* = 94): High-intensity statin alone without any PCSK9 inhibitor administration.

All patients received guideline-directed background medication including aspirin, P2Y12 inhibitor, nitrate, β-blocker, and RAAS inhibitor tailored to individual clinical conditions. No patients in either group received ezetimibe or other non-statin lipid-lowering agents during the 3-month follow-up period, ensuring that the observed lipid-lowering effects were attributable solely to the assigned statin-based regimens with or without evolocumab.

### 2.4 Observation endpoints

Baseline, discharge, 1-month, and 3-month follow-up data were collected for serum total cholesterol (TC), LDL-C, HDL-C, triglyceride (TG), lipoprotein(a) [Lp(a)], hs-CRP, liver/kidney biochemistry, and creatine kinase.

Primary endpoint: Composite 3-month MACE including cardiac death, non-fatal myocardial infarction, ischemic stroke, unplanned cardiac rehospitalization, and hospitalization-requiring recurrent angina.

Secondary endpoints: Individual components of MACE and all drug-related adverse events.

### 2.5 Follow-up protocol

All participants were followed via outpatient clinical revisit combined with standardized telephone interview at 1 and 3 months post-discharge. These telephone interviews are part of our institutional standard clinical care pathway for all high-risk acute coronary syndrome patients, conducted to monitor medication adherence, symptom control, and adverse events, rather than a research-specific procedure. Medication adherence, adverse drug reactions, and incident cardiovascular events were prospectively recorded from these routine clinical interactions.

### 2.6 Sample size estimation

Sample size was calculated *a priori* using PASS 15.0. Based on a two-sided significance level of 0.05 and power of 80%, and assuming a 30% absolute risk reduction in 3-month MACE (from 28% in controls to 12% in the evolocumab group, derived from our pilot data), at least 78 patients per group were required. Accounting for a 15% potential loss to follow-up, we aimed to enroll 90 patients per group. The final enrollment of 92 and 94 patients exceeded the minimum requirement.

### 2.7 Statistical analysis

All statistical analyses were performed with SPSS version 26.0 (IBM Corp., Armonk, NY, USA) and R version 4.3.1 (R Foundation for Statistical Computing, Vienna, Austria). Continuous data were expressed as mean ± standard deviation; categorical variables were presented as count (percentage). Missing data were minimal (<3% for all variables) and handled by multiple imputation with chained equations (MICE) using 20 imputed datasets.

#### 2.7.1 Inverse probability of treatment weighting (IPTW)

Although baseline characteristics were well-balanced between groups, we additionally performed IPTW as a sensitivity analysis to further minimize potential residual confounding. Propensity scores were calculated using a logistic regression model including age, sex, hypertension, diabetes, smoking, prior myocardial infarction, prior stroke, LVEF, SYNTAX score, baseline LDL-C, and hs-CRP. Balance was assessed using standardized mean differences (SMDs), with SMD < 0.1 indicating adequate balance.

#### 2.7.2 Longitudinal analysis

Repeated-measures ANOVA was performed with Greenhouse-Geisser correction applied when the sphericity assumption (Mauchly's test) was violated. *Post-hoc* pairwise comparisons were adjusted using Bonferroni correction.

#### 2.7.3 Survival analysis

Kaplan–Meier curves with Log-rank tests were used to compare cumulative MACE-free survival. Fine-Gray subdistribution hazard models were used to estimate the cumulative incidence of non-fatal MACE, treating cardiac death as a competing event. Results were reported as subdistribution hazard ratios (sHR) with 95% confidence intervals.

#### 2.7.4 Multivariate Cox regression

Variables with *P* < 0.2 in univariate screening, together with clinically relevant factors (age, diabetes, LVEF < 50%, SYNTAX score, baseline LDL-C, and hs-CRP), were entered into a multivariate Cox proportional hazards regression model using the enter method. Variance inflation factor (VIF) was calculated to exclude multicollinearity (VIF < 5 defined as no obvious collinearity).

#### 2.7.5 Safety analysis

Adverse event rates were compared using the *χ*^2^ test or Fisher's exact test (for 2 × 2 tables with expected cell counts < 5). A two-tailed *P* < 0.05 was considered statistically significant unless otherwise specified.

### 2.8 Data availability

The datasets generated and analyzed during the current study are available from the corresponding author upon reasonable request.

## 3 Results

### 3.1 Baseline clinical characteristics before and after IPTW

A total of 186 patients were enrolled (observation group: *n* = 92, control group: *n* = 94). Raw baseline demographic, comorbidity, cardiac function, angiographic, and laboratory indicators showed no significant intergroup differences (all *P* > 0.05). After IPTW adjustment, all baseline parameters remained well-balanced, with all SMDs < 0.1 (Table 1).

**Table 1:** Baseline characteristics before and after IPTW adjustment.

Index	Observation group (*n* = 92)	Control group (*n* = 94)	Raw SMD	Weighted SMD
Age, years	76.2 ± 8.5	75.7 ± 9.1	0.056	0.031
Male, *n* (%)	58 (63.0)	61 (64.9)	0.039	0.022
Hypertension, *n* (%)	79 (85.9)	82 (87.2)	0.038	0.019
Diabetes mellitus, *n* (%)	42 (45.7)	45 (47.9)	0.044	0.028
Smoking history, *n* (%)	35 (38.0)	38 (40.4)	0.049	0.025
Prior myocardial infarction, *n* (%)	28 (30.4)	31 (33.0)	0.055	0.030
Prior stroke, *n* (%)	19 (20.7)	21 (22.3)	0.039	0.021
LVEF, %	52.3 ± 9.7	51.8 ± 10.2	0.050	0.027
SYNTAX score	26.8 ± 4.2	27.1 ± 3.9	0.074	0.035
Baseline LDL-C, mmol/L	3.41 ± 0.76	3.38 ± 0.72	0.041	0.023
Baseline hs-CRP, mg/L	12.7 ± 5.8	13.1 ± 6.2	0.067	0.033

SMD, standardized mean difference; LVEF, left ventricular ejection fraction; LDL-C, low-density lipoprotein cholesterol; hs-CRP, high-sensitivity C-reactive protein.

### 3.2 Dynamic changes of serum lipids

Repeated-measures ANOVA demonstrated significant main effects of treatment, time, and treatment × time interaction for LDL-C, TC, and Lp(a) (all *P*_interaction < 0.001). Mauchly's test indicated violation of sphericity for LDL-C (*W* = 0.72, *P* = 0.003); thus, Greenhouse-Geisser corrected degrees of freedom were used. No significant interaction was detected for TG and HDL-C (*P* > 0.05).

At 3-month follow-up, the observation group achieved substantially lower TC, LDL-C, and Lp(a) vs. the control group. LDL-C reduction rate reached 61.3% in the observation group vs. 32.2% in the control group. The LDL-C target achievement rate (<1.8 mmol/L) was 89.1% in the observation group vs. 27.7% in the control group (*P* < 0.001) (Table 2).

**Table 2:** Lipid indicators at baseline and 3-month follow-up.

Parameter	Observation group (*n* = 92)	Control group (*n* = 94)	*P*-value
TC, baseline(mmol/L)	5.68 ± 1.03	5.62 ± 0.98	0.691
TC, 3 months(mmol/L)	3.12 ± 0.67	4.25 ± 0.89	<0.001
TG, baseline(mmol/L)	1.89 ± 0.75	1.85 ± 0.71	0.723
TG, 3 months(mmol/L)	1.52 ± 0.58	1.67 ± 0.64	0.098
HDL-C, baseline(mmol/L)	1.08 ± 0.25	1.06 ± 0.23	0.574
HDL-C, 3 months (mmol/L)	1.15 ± 0.27	1.11 ± 0.24	0.287
Lp(a), baseline, mg/L	326 ± 187	318 ± 179	0.765
Lp(a), 3 months, mg/L	178 ± 102	265 ± 143	<0.001

TC, total cholesterol; LDL-C, low-density lipoprotein cholesterol; HDL-C, high-density lipoprotein cholesterol; TG, triglycerides; Lp(a), lipoprotein(a).

### 3.3 Variation of hs-CRP inflammatory marker

Repeated-measures ANOVA confirmed a significant treatment × time interaction for hs-CRP (*P* < 0.001). Both groups presented gradual hs-CRP reduction during follow-up, while the declining magnitude was markedly larger in the evolocumab combination group: 48.7% reduction at 1 month vs. 26.1% in the statin monotherapy group (*P* < 0.001).

### 3.4 Three-month MACE and individual cardiovascular events

The total 3-month MACE rate was significantly lower in the observation group (11/92, 11.96%) vs. the control group (27/94, 28.72%; *P* = 0.004). Individual endpoint analysis showed significant reductions in non-fatal myocardial infarction, recurrent angina, and unplanned cardiac rehospitalization in the observation group (all *P* < 0.05); cardiac death showed no between-group statistical difference (*P* = 0.183) (Table 3).

**Table 3:** Three-month component MACE comparison [*n* (%)].

Outcome	Observation group (*n* = 92)	Control group (*n* = 94)	*P*-value
Total MACE	11 (11.96)	27 (28.72)	0.004
Cardiac death	1 (1.09)	4 (4.26)	0.183
Non-fatal myocardial infarction	2 (2.17)	8 (8.51)	0.047
Cardiac rehospitalization	5 (5.43)	14 (14.89)	0.026
Recurrent angina	3 (3.26)	11 (11.70)	0.028

**Figure 2:**
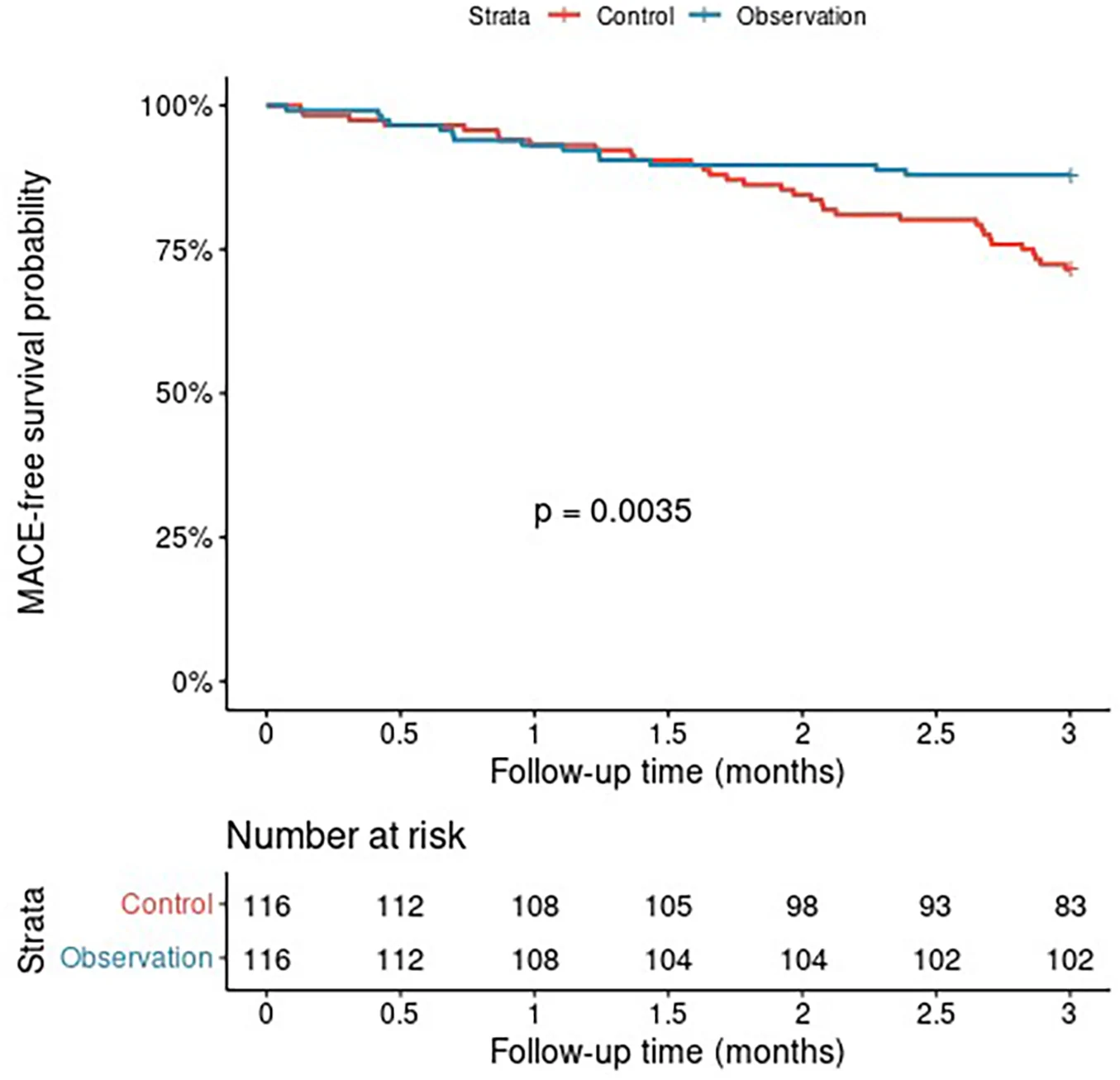
Kaplan–Meier survival curves for 3-month MACE-free survival in observation and control groups. Kaplan–Meier analysis showed that the 3-month MACE-free survival rate of the observation group was significantly higher than that of the control group (88.04% vs. 71.28%, Log-rank *χ*^2^ = 8.72, *P* = 0.003). Fine-Gray competing risk analysis confirmed the robustness of the primary finding (sHR for non-fatal MACE = 0.44, 95% CI: 0.26–0.74, *P* = 0.002).

Kaplan–Meier analysis showed that the 3-month MACE-free survival rate of the observation group was significantly higher than that of the control group (88.04% vs. 71.28%, Log-rank *χ*^2^ = 8.72, *P* = 0.003) (Figure 2). Fine-Gray competing risk analysis confirmed the robustness of the primary finding (sHR for non-fatal MACE = 0.44, 95% CI: 0.26–0.74, *P* = 0.002).

### 3.5 Multivariate Cox regression analysis

**Figure 3:**
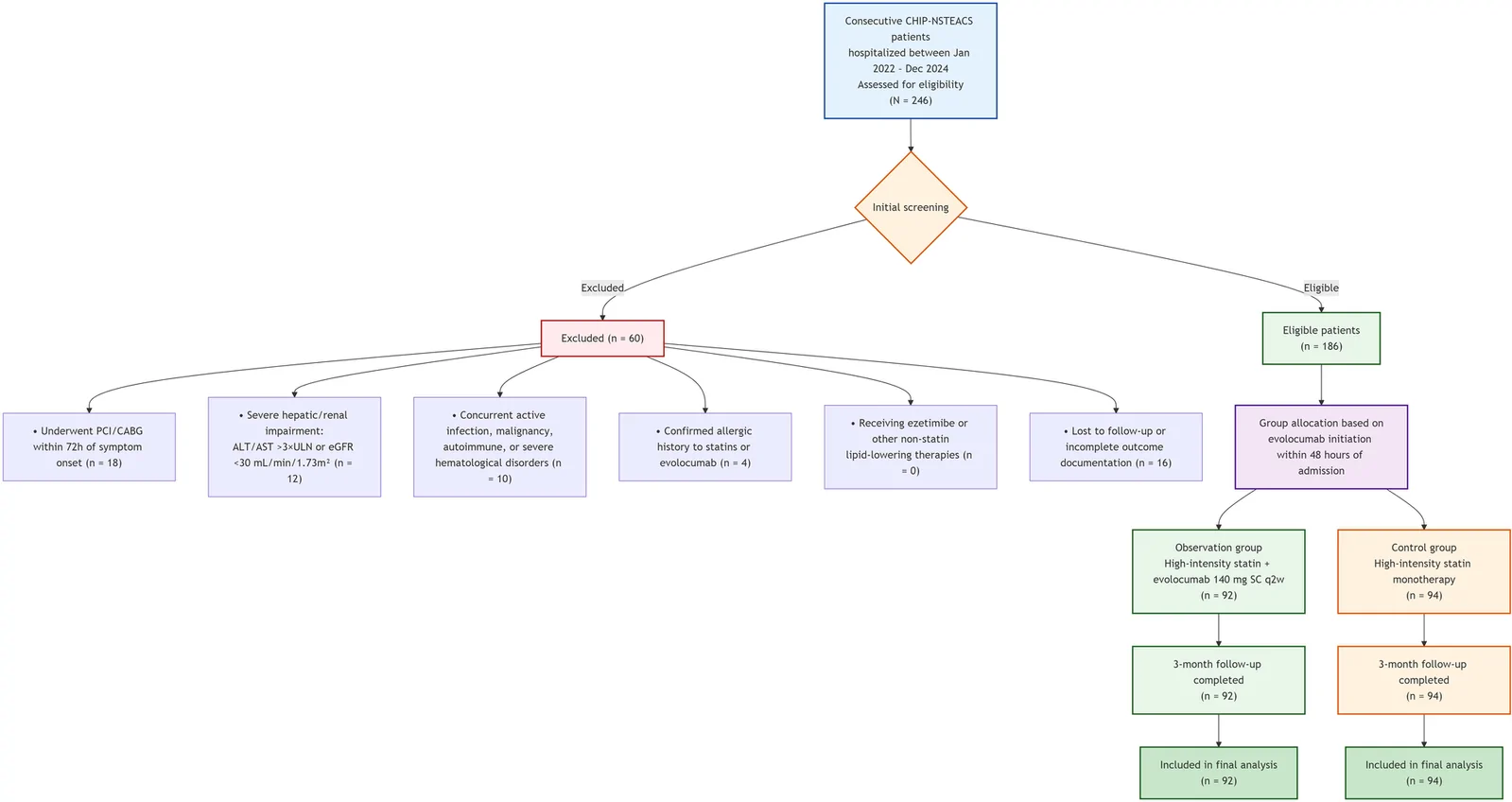
Forest plot of multivariate Cox regression analysis for independent predictors of 3-month MACE. The vertical dashed line indicates HR = 1.0. HR < 1 represents a protective factor, and HR > 1 represents a risk factor. MACE, major adverse cardiovascular events; HR, hazard ratio; CI, confidence interval; LVEF, left ventricular ejection fraction; LDL-C, low-density lipoprotein cholesterol; hs-CRP, high-sensitivity C-reactive protein.

No obvious multicollinearity was observed among covariates (all VIF < 5). In univariate Cox regression, early evolocumab (HR = 0.41, 95% CI: 0.24–0.69, *P* = 0.001), diabetes (HR = 1.58, 95% CI: 0.99–2.52, *P* = 0.054), and LVEF < 50% (HR = 1.92, 95% CI: 1.15–3.21, *P* = 0.013) were associated with 3-month MACE (*P* < 0.2). After adjusting for age, diabetes, LVEF < 50%, SYNTAX score, baseline LDL-C, and hs-CRP, early evolocumab treatment independently reduced 3-month MACE risk (HR = 0.42, 95% CI: 0.25–0.71, *P* = 0.001). Diabetes mellitus (HR = 1.63, 95% CI: 1.01–2.64, *P* = 0.045) and LVEF < 50% (HR = 1.87, 95% CI: 1.12–3.12, *P* = 0.017) were independent adverse prognostic factors (Table 4, Figure 3).

**Table 4:** Multivariate Cox regression for predictors of 3-month MACE.

Variable	HR	95%CI	*P*-value
Early evolocumab treatment	0.42	0.25–0.71	0.001
Age (per 1 year increase)	1.35	0.94–1.93	0.102
Diabetes mellitus	1.63	1.01–2.64	0.045
LVEF < 50%	1.87	1.12–3.12	0.017
SYNTAX score (per 1 point increase)	1.21	0.88–1.66	0.245
Baseline LDL-C (per 1 mmol/L increase)	1.29	0.91–1.83	0.156
Baseline hs-CRP (per 1 mg/L increase)	1.18	0.85–1.64	0.312

### 3.6 Safety assessment

All adverse reactions were mild and well-tolerated; no fatal adverse events, severe rhabdomyolysis, or irreversible hepatic/renal damage occurred. Injection-site reaction was exclusive to the evolocumab group. Mild myalgia and transient liver enzyme elevation were sporadically observed in both cohorts. The total adverse event rate was comparable between groups (5.43% vs. 4.26%, *P* = 0.715) (Table 5).

**Table 5:** Between-group adverse event comparison [*n* (%)].

Adverse event	Observation group (*n* = 92)	Control group (*n* = 94)	*P*-value
Injection site reaction	3 (3.26)	0 (0.00)	0.078
Mild myalgia	2 (2.17)	3 (3.19)	0.674
Transient liver enzyme elevation	0 (0.00)	1 (1.06)	0.323
Severe hepatic/renal dysfunction	0 (0.00)	0 (0.00)	–
Rhabdomyolysis	0 (0.00)	0 (0.00)	–
Severe allergic reaction	0 (0.00)	0 (0.00)	–
Total adverse events	5 (5.43)	4 (4.26)	0.715

## 4 Discussion

Non-revascularized CHIP-NSTEACS represents the highest-risk subset within the ACS spectrum, characterized by diffuse multivessel coronary lesions, severe vascular calcification, advanced age, and multiple systemic comorbidities (9, 10). Deprived of mechanical revascularization to ameliorate myocardial ischemia, clinical outcomes of this population heavily rely on optimized medical management including intensive lipid modulation and anti-inflammatory therapy (11). Standard high-intensity statin monotherapy frequently fails to achieve guideline LDL-C targets, cannot effectively lower genetically determined Lp(a), or suppress persistent low-grade vascular inflammation, resulting in excessive short-term recurrent ischemia and unplanned readmission (12). The present IPTW-adjusted retrospective cohort study confirmed that early in-hospital evolocumab combined with statin produces robust lipid-lowering and anti-inflammatory effects, reduces 3-month composite MACE without increasing drug-related adverse events, providing high-quality real-world evidence for pharmacotherapy optimization in non-revascularized CHIP-NSTEACS.

### 4.1 Lipid-modulating effects

Our research validated superior lipid-modulating capacity of early evolocumab in ultra-high-risk CHIP-NSTEACS. PCSK9 inhibitors block hepatic LDL receptor degradation and synergize with statin to accelerate circulating LDL-C clearance (7). After 3-month treatment, LDL-C reduction reached 61.3% in the combination arm with 89.1% target attainment rate, far exceeding statin-only group. Notably, evolocumab yielded meaningful Lp(a) reduction, a residual inherited cardiovascular risk factor refractory to statin monotherapy (13). Lp(a) drives atherosclerotic plaque progression, thrombosis formation, and plaque destabilization; simultaneous LDL-C and Lp(a) lowering impedes diffuse coronary lesion progression specifically for CHIP patients with extensive atherosclerotic burden (14). These lipid findings align with landmark FOURIER and ODYSSEY OUTCOMES RCT results and extend existing evidence to non-revascularized CHIP populations excluded from most large-scale randomized trials (7, 8).

### 4.2 Anti-inflammatory effects

This study demonstrated evolocumab's independent anti-inflammatory pleiotropy beyond lipid reduction, reflected by prominent hs-CRP decrement (15). Elevated hs-CRP correlates closely with vulnerable plaque rupture and recurrent ischemic events, and non-revascularized CHIP-NSTEACS remains in sustained systemic inflammatory overactivation due to extensive unstable plaque load (16). A 48.7% hs-CRP decline at 1 month in the evolocumab group indicated direct anti-inflammatory properties of PCSK9 inhibition. Preclinical studies confirm that PCSK9 participates in endothelial activation, macrophage infiltration, and pro-inflammatory cytokine release; PCSK9 blockade suppresses foam cell generation, stabilizes plaque fibrous cap, and lowers plaque rupture risk (17). For patients unable to receive invasive revascularization, pharmacological plaque stabilization via early evolocumab partially compensates for lost mechanical revascularization benefit and shortens the early recurrent ischemia risk window.

### 4.3 Clinical outcomes

Multivariate Cox regression verified early evolocumab as an independent protective factor for short-term MACE reduction after rigorous confounder adjustment. The 3-month total MACE was reduced by approximately 58% in the evolocumab group, predominantly driven by fewer non-fatal MIs, recurrent angina, and cardiac rehospitalizations, consistent with its dual lipid-lowering and anti-inflammatory mechanisms. Fine-Gray competing risk analysis, treating cardiac death as a competing event, confirmed the robustness of this finding (18). Current international lipid guidelines prioritize PCSK9 inhibitor application for post-PCI ACS patients, while formal recommendations are absent for non-revascularized CHIP cohorts (19). Our real-world data fill this critical evidence gap and support early intensive evolocumab-based lipid-lowering strategies for inoperable CHIP-NSTEACS.

### 4.4 Comparison with previous studies

The magnitude of LDL-C reduction (61.3%) in our study is consistent with the FOURIER trial (59% at 48 weeks) and the EVOPACS study (64.2% at 8 weeks), despite our population being older and entirely non-revascularized (7, 20). Notably, the absolute MACE reduction (16.8% absolute risk reduction over 3 months) appears larger than that reported in FOURIER (1.5% over 2.2 years), likely reflecting the ultra-high baseline risk of our cohort and the shorter follow-up window capturing early events (21). This observation supports the concept that patients with the highest baseline risk derive the greatest absolute benefit from intensive lipid lowering—a “risk-benefit paradox” that has important clinical implications for resource allocation.

### 4.5 Safety and clinical implications

Safety data corroborated favorable tolerability of evolocumab among elderly multi-comorbid CHIP patients. Mild injection-site discomfort and occasional myalgia were the only evolocumab-related adverse events without severe organ toxicity, matching global multicenter safety datasets from pivotal PCSK9 inhibitor trials (22). Satisfactory safety facilitates long-term continuous medication in fragile elderly high-risk populations. Our findings have several practical implications: (1) For patients deemed inoperable due to prohibitive surgical risk, early evolocumab initiation (within 48 h of admission) should be considered as part of standard care; (2) The rapid onset of action (within 1 month) suggests that even short-term treatment may provide meaningful event reduction; (3) The favorable safety profile supports use in elderly patients with polypharmacy.

### 4.6 Study limitations

Several limitations should be acknowledged. First, the single-center retrospective nature inherently carries residual unmeasured confounding despite IPTW adjustment; multicenter prospective trials are required for external validation. Second, the limited 3-month follow-up precludes evaluation of long-term (≥12 months) cardiovascular endpoints. Third, intracoronary imaging modalities (OCT/IVUS/CCTA) were unavailable to directly quantify *in-vivo* plaque morphological changes. Fourth, data on concomitant anti-inflammatory agents (colchicine, etc.) were not collected for subgroup correction. Future prospective research should incorporate serial coronary imaging and extended long-term follow-up.

## 5 Conclusion

For non-revascularized CHIP-NSTEACS patients, early evolocumab add-on therapy based on high-intensity statin rapidly reduces LDL-C and Lp(a), suppresses systemic inflammation, stabilizes vulnerable atherosclerotic plaque, and significantly decreases 3-month MACE,non-fatal myocardial infarction, and unplanned cardiac readmission with a favorable safety profile. Early evolocumab combination is a valuable intensive pharmacological alternative for this ultra-high-risk cohort, providing real-world data to refine future clinical guideline recommendations.

## Data Availability

The raw data supporting the conclusions of this article will be made available by the authors, without undue reservation.
